# Molecular Regulation of Bone Marrow Metastasis in Prostate and Breast Cancer

**DOI:** 10.1155/2014/405920

**Published:** 2014-07-23

**Authors:** Fakher Rahim, Saeideh Hajizamani, Esmaeil Mortaz, Ahmad Ahmadzadeh, Mohammad Shahjahani, Saeid Shahrabi, Najmaldin Saki

**Affiliations:** ^1^Health Research Institute, Ahvaz Jundishapur University of Medical Sciences, Ahvaz 61357-15794, Iran; ^2^Health Research Institute, Research Center of Thalassemia & Hemoglobinopathy, Ahvaz Jundishapur University of Medical Sciences, Ahvaz 61357-15794, Iran; ^3^Division of Pharmacology and Pathophysiology, Utrecht Institute for Pharmaceutical Sciences, Faculty of Sciences, Utrecht University, 80082 Utrecht, The Netherlands; ^4^Clinical Tuberculosis and Epidemiology Research Center, National Research and Institute of Tuberculosis and Lung Diseases (NRITLD), Shahid Beheshti University of Medical Sciences, 19575/154 Tehran, Iran; ^5^Cell and Molecular Biology Group, Airways Disease Section, National Heart and Lung Institute, Imperial College London, Dovehouse Street, London SW7 2AZ, UK; ^6^Department of Biochemistry and Hematology, Faculty of Medicine, Semnan University of Medical Sciences, Semnan 35131-38111, Iran

## Abstract

Metastasis is a multistep process, which refers to the ability to leave a primary tumor through circulation toward the distant tissue and form a secondary tumor. Bone is a common site of metastasis, in which osteolytic and osteoblastic metastasis are observed. Signaling pathways, chemokines, growth factors, adhesion molecules, and cellular interactions as well as miRNAs have been known to play an important role in the development of bone metastasis. These factors provide an appropriate environment (soil) for growth and survival of metastatic tumor cells (seed) in bone marrow microenvironment. Recognition of these factors and determination of their individual roles in the development of metastasis and disruption of cellular interactions can provide important therapeutic targets for treatment of these patients, which can also be used as prognostic and diagnostic biomarkers. Thus, in this paper, we have attempted to highlight the molecular regulation of bone marrow metastasis in prostate and breast cancers.

## 1. Introduction 

Metastasis refers to the ability to leave a primary tumor through circulation toward the distant tissue and form a secondary tumor. Metastasis involves five steps, including local invasion and migration through extracellular matrix and surrounding stromal cells, intravasation to blood capillaries, survival in circulation, extravasation, colonization, and proliferation in the distal tissue [1]. An environment rich in growth factors, cytokines, chemokines, and signaling molecules for survival and growth of tumor cells is provided by a metastatic niche. This is known as “Paget seed and soil” theory and states that tumor metastasis entails a series of interactions between the tumor cells and stromal cells [2, 3]. Disrupting these reactions can serve as a therapeutic intervention for bone metastasis.

Bone marrow (BM) microenvironment includes osteoblastic (endosteal) and vascular niches, which provide an environment to support hematopoietic and nonhematopoietic stem cells such as mesenchymal stem cells [4]. In a normal niche, BM microenvironment consists of such stromal cells as osteoblasts (OB) and nonstromal cells like osteoclasts (OCL), which play an important role in bone remodeling and niche structure [4, 5]. Bone homeostasis is maintained by a balanced production of OB and OCL. Disruption of this balance due to the presence of cancer cells converts normal niche to cancerous or metastatic niche [6, 7].

There are two types of bone marrow tissue, including red and yellow marrows. Red marrow contains hematopoietic stem cells (HSC) and yellow marrow mainly consists of fat cells [8]. Bone marrow, especially red marrow, is a common site of metastasis. Excessive blood flow in red marrow, presence of adhesion molecules on tumor cells binding stromal BM cells, and production of angiogenic and bone-resorbing factors enhancing tumor growth are among the factors causing bone metastasis [2]. Therefore, it can be stated that BM environment has unique biological properties for homing, survival, and proliferation of circulating cancer cells. Cancer cells are capable of taking advantage of these unique properties to colonize the bone, ultimately causing bone destruction and disruption of the normal function of bone [9]. Bone metastasis takes the osteolytic or osteoblastic forms [10]. In this review, the role of several cellular signaling pathways, cytokines, chemokines, and adhesion molecules, providing proper circumstances for BM metastasis in breast and prostate cancers, will be discussed (Table 1). Moreover, miRNA modifications during metastasis will be further highlighted.

## 2. Molecular Mechanism of Osteolytic Bone Metastasis

**Figure 1:**
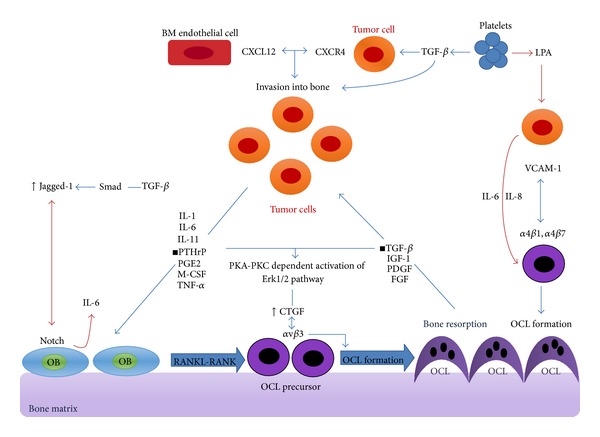
The mechanism of bone resorption in bone marrow metastasis. Tumor cells highly express CXCR4, causing their homing to BM by CXCL12/CXCR4 signaling. Metastatic tumor cells in bone release PTHrP, which induces OCL formation and bone resorption. Bone resorption releases the growth factors stored in bone such as TGF-*β*, IGF-1, PDGF, and FGF. TGF-*β* increases the expression of PTHrP, IL-1, IL-6, IL-8, IL-11, PGE2, M-CSF, and TNF-*α* by direct impact on cancer cells. These factors induce OCL formation by increasing RANKL expression on OB cells. RANKL binds to RANK on OCL precursor. The expression CTGF/CCN2 gene is triggered by PTHrP and TGF-*β* released by tumor cells through PKA-PKC-dependent activation of ERK1/2 pathway. TGF-*β* also increases jagged-1 expression in tumor cell through the Smad pathway. Cancer cells expressing jagged-1 activate Notch signaling in OB, stimulating the secretion of IL-6, which stimulates tumor cell growth. VCAM-1 is expressed in tumor cells and binds *α*4*β*7 and *α*4*β*1 integrins on OCL progenitors, causing OCL differentiation. Platelet-derived TGF-*β* and direct contact between platelets and tumor cells increase invasion and metastasis. platelet-derived lysophosphatidic acid (LPA) induces the release of IL-6 and IL-8 from tumor cells, which eventually leads to osteoclastic activation and bone resorption. Abbreviations: BM, bone marrow; CXCL12, C-X-C motif chemokine 12; CXCR4, C-X-C chemokine receptor 4; TGF-*β*, transforming growth factor *β*; LPA, lysophosphatidic acid; PTHrP, parathyroid hormone-related protein; PGE2, prostaglandin E-2; M-CSF, macrophage colony stimulating factor, TNF-*α*, and tumor necrosis factor *α*; IGF-1, Insulin-like growth factor; PDGF, platelet-derived growth factor; FGF, fibroblast growth factor; VCAM-1, vascular-endothelial molecule-1; CTGF, connective tissue growth factor; RANKL, receptor activator of nuclear factor-*κ*B ligand; OCL, osteoclast; OB, osteoblast.

It has been established that 65–75% of breast cancer patients are encountered with osteolytic bone metastasis [11]. Osteolytic bone metastasis occurs during a vicious cycle between tumor and BM cells, in which bone-derived transforming growth factor *β* (TGF-*β*) and tumor-derived parathyroid hormone-related protein (PTHrP) cause osteolytic bone metastasis [12] (Figure 1). Tumor cells (including human breast cancer cells) release PTHrP, which induces OCL formation and bone resorption by affecting osteoclast precursors [13]. Bone resorption releases growth factors stored in bone matrix such as TGF-*β*, insulin-like growth factor 1 (IGF-1), platelet-derived growth factor (PDGF), and fibroblast growth factor (FGF) [14]. TGF-*β* increases the expression of PTHrP and other growth factors like IL-1, IL-6, IL-8, IL-11, prostaglandin E-2 (PGE2), macrophage colony stimulating factor (M-CSF), and tumor necrosis factor *α* (TNF-*α*) by direct impact on cancer cells, resulting in enhanced tumor growth in BM [7, 15]. Symptoms like bone pain, hypercalcemia, fracture, and spinal cord compression appear in this type of metastasis [16]. The effect of factors released by tumor cells on OCLs is mediated by receptor activator of nuclear factor-*κ*B (NF-*κ*B) ligand (RANKL), which is secreted by bone marrow mesenchymal stem cells [5]. PTHrP, PGE2, IL-1, and IL-6 induce OCL formation by increasing RANKL expression in stromal and immature OB cells. In addition, RANKL induces OCL formation and increases OCL survival by binding RANK on OCL precursors in NF-*κ*B, Jun N-terminal kinase (JNK), Erk1/2, and P38/MAP kinase signaling pathways [15, 17]. The expression of connective tissue growth factor (CTGF/CCN2) gene is triggered in BM by PTHrP and TGF-*β* released by breast cancer cells through PKA-PKC-dependent activation of the ERK1/2 pathway, augmenting osteoclastogenesis by binding *α*v*β*3 integrin on OCL progenitors [12]. CTGF and IL-11 are targets of TGF-*β* signaling, and their increased expression in breast cancer MDA-MB-231 cell line results in enhanced osteolytic bone metastases [18]. TGF-*β* induces Smad2/3 and Smad4 binding to the promoter of IL-11 gene, and CTGF induction by TGF-*β* is a Smad-dependent process [18]. Bone morphogenetic protein (BMP) signaling promotes bone invasion and metastasis in breast cancer through the Smad pathway, and inhibition of any of these pathways will inhibit metastasis. Phosphorylation of R-Smads and accumulation of phosphorylated Smads in the nucleus are an index of TGF-*β* and BMP stimulation [19]. Thus, breast cancer metastasis to bone disrupts the balance towards OCL activity.

### 2.1. CXCL12/CXCR4 Signaling in BM Metastatic Breast Cancer

Chemokines such as CXCL12/CXCR4 form a superfamily of cytokines that regulate cell migration and play an important role in the regulation of metastasis [20]. CXCR4 (C-X-C chemokine receptor 4) is highly expressed in breast cancer cells, and its ligand (C-X-C motif chemokine 12, CXCL12) is expressed in high levels in tissues invaded by metastasis [11]. Bone metastatic breast cancer cells express activated Src, which is necessary for Akt activation and cell survival in response to CXCL12 and to resist the effects of proapoptotic signal by TRAIL [21, 22]. This means that CXCL12 binds to CXCR4 and leads to the activation of Akt, for which Src is required. ErbB2 signaling increases CXCR4 translation through activation of PI3 kinase/Akt/mTOR pathway and decreases CXCR4 degradation. Synergy between ErbB2 and CXCR4 seems to enhance the ability of breast cancer cells to metastasize to different sites [11].

### 2.2. Adhesive Interactions in BM Metastatic Breast Cancer

Interaction between tumor cells and bone marrow stromal cells is critical for colonization in distant target tissues. Vascular-endothelial molecule-1 (VCAM-1) is expressed in breast cancer cells by ectopically expressed NF-*κ*B, which mediates this interaction. VCAM-1 binds *α*4*β*7 and *α*4*β*1 (VLA-4) integrins on OCL progenitors with high affinity, causing OCL differentiation and osteoclastogenesis [23, 24]. *α*4 or VCAM-1 blocking antibodies effectively inhibit bone metastasis [24]. *α*v*β*3 integrin is expressed in OCL and plays an important role in OCL attachment to bone and in bone reabsorption. High levels of this protein have been observed in MDA-MB231 cells [25]. High levels of CD44 expression on breast cancer cells promote invasion and adhesion to BM endothelial cells. CD44 binding to hyaluronan and its activation leads to IL-8 production in the tumor cell, which stimulates osteolysis [26].

### 2.3. Jagged-1/Notch Signaling in BM Metastatic Breast Cancer

Notch ligand jagged-1 (JAG1) is highly expressed in bone metastatic tumor cells and is again activated by bone-derived TGF-*β* during osteolytic bone metastasis [27]. Cancer cells expressing jagged-1 activate Notch signaling in OB and stimulate the secretion of IL-6 [29]. IL-6 in turn affects tumor cells, stimulating their growth and resistance to chemotherapy. Jagged-1 expression activates OCL differentiation, resulting in bone reabsorption [22, 28, 29]. Bone reabsorption induces the release of TGF-*β* from bone matrix, increasing jagged-1 expression in tumor cell through the Smad pathway, with impaired Notch signaling pathway in the bone microenvironment reducing bone metastasis [27]. Tumor cells expressing jagged-1 may indirectly alter the expression of OB-derived RANKL and osteoprotegerin (OPG) [27].

### 2.4. Other Factors Involved in BM Metastatic Breast Cancer

The expression of preprotachykinin-1 (PPT-1) is increased in breast cancer cell lines and in biopsy samples of malignant breast compared with normal mammary cells and together with its receptors (i.e., neurokinin-1 (NK-1)) and neurokinin-2 (NK-2) is associated with homing, integration, dysfunction in BM microenvironment, and eventual metastasis [33]. Overexpression of colony stimulating factor (CSF-1) in breast cancer leads to development of bone metastasis. Metastatic tumor cells express both secreted and surface forms of CSF-1. The surface form by itself induces the differentiation and survival of OCL, protecting OCL against the inhibitory effect of TGF-*β*. CSF-1 expression in metastatic tumor cells can stimulate OCL activity and can enhance osteolysis in breast cancer metastasis [32]. The expression of bone marrow stromal protein 2 (BST2), which can be associated with the development of bone metastasis in human breast cancer, is significantly increased in bone metastatic breast cancer cell lines and tumor tissue compared with nonbone metastatic breast cancer cell lines. It can also be used as a novel biomarker in metastasized breast cancers [45]. IL-11 and granulocyte macrophage-colony stimulating factor (GM-CSF) are target genes of Runx2/CBF*β* as OCL activators in breast cancer cells [30]. Runx2/CBF*β* mediates inhibition of OB differentiation in MDA-MB-231 cells by inducing sclerostin as an antagonist of Wnt signaling. Sclerostin functions as Dickkopf homolog 1 (DKK1) in MM and is involved in osteolytic metastasis by inhibiting Wnt signaling and OB differentiation [30, 46]. GM-CSF is also a key target for NF-*κ*B, and increased expression of it in breast cancer is associated with NF-*κ*B activity. Therefore, NF-*κ*B can be used as a target for treatment of breast cancer and prevention of metastasis [47]. DKK1 is frequently upregulated in human breast cancer tissue and in metastatic cancer cells and is involved in development and progression of osteolytic metastasis. Breast cells produce high levels of DKK1 by increasing the activity of Wnt/*β*-catenin signaling, by inhibiting differentiation of OB and decreasing the expression of OPG and RANKL [31]. DKK1 can be a potential therapeutic target in treatment of metastasis in breast cancer. As an antagonist of Wnt/*β*-catenin, DKK is released from tumor cells, playing an important role in creating a link between breast cancer cell and osteolytic bone metastasis [31]. Thus, by stimulation of DKK-1 expression in tumor cells, IL-6 inhibits Wnt-mediated osteogenesis, causing an imbalance in bone homeostasis and increased bone degradation [17].

## 3. Molecular Mechanism of Osteoblastic Bone Metastasis

Formation and activity of OB are increased in patients with osteoblastic bone metastasis. Nearly, 70% of patients with prostate cancer (PCa) have bone metastases at the end stages. PCa cells preferentially invade and home to OB niche in BM [36] and cause osteoblastic metastasis by releasing osteoblast-promoting factors such as BMP, Wnt family ligand, endothelin-1, and PDGF. These are associated with increased bone density and bone marrow displacement, but many of the patients have osteolytic metastasis as well [2, 6, 7]. In coculture of PCa cell lines MDA-PCa2a and MDA-PCa 2b with mouse OB cells, growth, differentiation, and differentiation markers of OB are induced through increased Cbf*α*1, procollagen-type1, osteocalcin, and osteopontin during the biological process [48]. Endothelin-1 plays an important role in stimulation of OB proliferation, differentiation, and bone formation and is increased in the serum of patients with PCa metastasized to bone [15]. Endothelin-1 increases the activity of OB by inhibiting DKK-1 expression in marrow stromal cells [34]. Besides, Wnt signaling pathway has an important role in osteoblastic metastasis. Thus, it promotes the proliferation, activity, and survival of OBs [49]. Moreover, it increases the expression of type 1 collagen in OB, which is a protein constituent of bone matrix [35]. The existence of osteoblastic metastases can be confirmed by increased alkaline phosphatase (ALP) levels in the serum [2].

### 3.1. CXCL12/CXCR4 Signaling in BM Metastatic Prostate Cancer

CXCL12/CXCR4 is an essential signal produced by stem cell niche to regulate HSC. CXCL12 (SDF-1) is highly expressed on OB, endothelial cells, and stromal cells in BM and is involved in regulation of HSC quiescence and homing. PCa cells highly express CXCR4, which causes their homing to BM by CXCL12/CXCR4 signaling, competing with HSC to settle and stay in the niche [36]. The absence of tumor suppressor phosphatase and tensin homolog (PTEN) in PCa cell line (DU145) and subsequent activation of PI3K/Akt pathway, which frequently occurs in PCa, lead to an increase in CXCR4 expression, regulating the growth and metastasis of bone through CXCL12/CXCR4 pathway. CXCR4 expression in human PCa is associated with poor survival [20]. Akt inhibitors may potentially be used as anticancer agents to target metastasis in PCa [20]. The level of Treg cells is higher in patients with BM metastatic PCa than in patients without BM metastasis [50]. Treg cells express high levels of CXCR4, which ultimately leads to the Treg cell migration to BM through CXCl12/CXCR4 pathway. BM dendritic cells (DC) express high levels of RANK, which leads to Treg activation and pathological expansion through RANKL-RANK signaling. Thus, Treg cells are able to inhibit differentiation and function of OCL (mediated by activated T cell and M-CSF). This mechanism has been observed with reduced bone mineral density in mouse models of human prostate cancer [50].

### 3.2. Osteonectin as a Chemoattractive Factor in BM Metastatic Prostate Cancer

Osteonectin is a protein in the bone that binds collagen in a calcium-dependent manner, and its expression is increased in metastatic sites [51]. Osteonectin in bone promotes migration and invasion capacity of metastasizing PCa cells, including PC-3, and acts as a chemoattractive factor [37]. Matrix metalloprotease (MMP) activity, especially MMP2, which is associated with invasion and metastatic potential in cancer cells, is induced by osteonectin in human prostate and breast cancers [37]. S-ErbB3 stimulates the bone to secrete osteonectin, which subsequently enhances the invasion of PC-3 PCa cells by interacting with *α*v*β*3 and *α*v*β*5 cell surface receptors [38, 39]. The link between increased expression of sErbB3 and longer time to bone metastasis suggests that ErbB3 is involved in PCa progression into bone [52].

### 3.3. Sonic Hedgehog (Shh) Signaling in BM Metastatic Prostate Cancer

The role of Sonic hedgehog (Shh) and its signaling pathway components (which are overexpressed in disease progression and metastasis) has been reported in PCa. Expression of Shh in PCa cells induces differentiation of preosteoblasts through a Gli1-dependent mechanism [29, 40]. Ascorbic acid (AA) upregulates paracrine Shh signaling in MC3T3 preosteoblasts. Matrix collagen is formed by OB in the presence of AA, potentiating Shh signaling between PCa cells and OBs and inducing OB differentiation [41].

### 3.4. Other Factors Involved in BM Metastatic Prostate Cancer

Increased production of urokinase-type plasminogen activator (u-PA) in PCa cells increases metastasis to bone [15]. Plasma levels of uPA and its receptor uPAR are significantly increased in patients with PCa compared to healthy subjects. UPA is associated with aggressive disease phenotype, progression, and metastasis to bone and can be used as a factor in the prognosis and progression of PCa [43, 44]. There is overexpression of uPA and uPAR in neuroblastoma, and their increased expression is associated with invasion, metastasis, and poor prognosis for neuroblastoma [53]. Katanin p60 is ectopically expressed during PCa progression into bone, and its increased expression may be involved in metastasis of cancer cells through stimulatory effect on cell motility [54]. NF-*κ*B is an important transcription regulator in PCa cells. PCa cells which have capacity to growth in the microenvironment of bone have higher NF-*κ*B activity, which upregulates the genes related to osteoclastogenesis such as GM-CSF, RANKL, uPA, and PTHrp but has no effect on proliferation of OB [55]. PCa cell binding to OB in hematopoietic stem cell niche induces the expression of TANK binding kinase 1 (TBK1) in PCa cells. TBK1 inhibits the mammalian target of rapamycin (mTOR) signaling pathway, and this induces dormancy and drug resistance in PCa cells. TBK1 enhances PCa stem-like cells and drug resistance in PCa. Rapamycin induces cell cycle arrest as an inhibitor of mTOR signaling, increasing resistance to chemotherapy in PCa cells [42]. Metastatic PCa cells can produce high levels of OPG (an inhibitor of RANKL) as well as a variety of other factors like PTHrP, M-CSF, TGF-*β*, uPA-plasmin, matrix metalloproteinases (MMP2 and 9), and interleukins 1 and 6 [7, 55].

## 4. The Role of Platelets in BM Metastasis

It has been shown that platelets, which are transient cells in BM microenvironment, are important for metastasis of a variety of solid tumors (Figure 1). Platelets bind circulating tumor cells, protecting them against anoikis (a type of programmed cell death occurring due to detachment of the cell from surrounding ECM) as well as against the innate immune system [2, 56, 57]. Platelet-derived TGF-*β* and direct contact between platelets and tumor cells synergistically activate TGF-*β*/Smad and NF-*κ*B pathways, leading to epithelial-mesenchymal transition (EMT), increased invasion, and metastasis [58]. In addition, during platelet aggregation by breast cancer cells, platelet-derived lysophosphatidic acid (LPA) induces the release of IL-6 and IL-8 from breast cancer cells, which eventually lead to osteoclastic activation and bone resorption [59]. Megakaryocyte ploidy is significantly higher in patients with metastatic disease. Megakaryocyte/platelet surface integrin *α*IIb/*β*3 may be involved in tumor colonization in bone marrow, since the mice lacking *β*3 integrin or those receiving *α*IIb/*β*3 inhibitors are protected against bone metastases [60].

## 5. MiRNAs and BM Metastasis

MiRNAs are small 19–22 nucleotide RNA molecules involved in regulation of processes such as proliferation and apoptosis [72]. Altered expression of miRNAs has been found to affect the mentioned cellular processes and may be directly related to cancer development and progression, ultimately resulting in metastasis [73]. miRNAs have been recognized as activators (metastamir) or suppressors of metastasis progression, and they are involved in various stages of metastasis [74] (Table 2). The expression of miR-16 in human PCa is decreased compared with normal prostate tissues, and evaluation of cellular models has shown that miR-16 inhibits prostate tumor growth through expression regulation of such genes as cyclin-dependent kinase 1 (CDK1) and CDK2, which are involved in controlling the cell cycle and proliferation [61]. Patients with metastatic PCa have a significantly lower expression of miR-143 and miR-145 compared with patients without metastasis. Upregulation of miR-143 and miR-145 decreases the invasive capacity of PC-3 cells in vitro and in vivo. EMT is suppressed through inhibition of mesenchymal markers vimentin and fibronectin and increased E-cadherin [63]. Increased expression of miRNA-143 and -145 inhibits cell viability and colony formation in bone metastasis of PC-3 cells isolated from PCa. Moreover, these miRNAs suppress tumor cell formation, expression of cancer stem cells (CSC) markers, and stemness factors such as CD133, CD44, Oct4, C-Myc, and K1f4 in PC-3 cells, ultimately preventing bone invasion and tumorigenicity [64]. Stemness is described as a pattern of gene expression that is common among all stem cells and distinguishes them from ordinary cells [75]. Human enhancer of filamentation 1 (HEF1) gene is a target of miR-145, and its expression is negatively correlated with miR-145 in primary PCa and bone metastasis. HEF1 expression is associated with an elevated level of PSA [65]. MiR-203 expression in bone metastatic PCa is significantly attenuated compared to normal tissue, and its reexpression suppresses metastasis in PCa in vitro. Indeed, miR-203 is an antimetastatic miRNA. Ectopic expression of miR-203 leads to repression of Runx2 and Smad4 [66]. Runx2 and Smad4 are critical in regulating the expression of genes involved in bone formation and are ectopically expressed in bone metastases and tumors [76]. The serum level of miR-141 is increased in patients with bone metastatic PCa and is related to metastatic lesion of the bone. A correlation has been reported between increased serum levels of miR-141 and the level of ALP but not that of PSA [62]. As a result, the serum miRNA level can function as a new biomarker for diagnosis and assessment of metastasis. Expression of miRNAs -508-5p, -145, -143, 33a, and -100 in bone metastasis is severely decreased compared with primary tumors of prostate [77]. MiR-218 increases the Wnt activity and abnormal expression of OB genes by downregulating three inhibitors of this pathway, including Sclerostin (SOST), DKK2, and secreted frizzled related-protein 2 (SFRP2) during osteogenesis, which participate in the homing and growth of metastasized cells into the bone [67]. MiR-218 expression is stimulated in response to Wnt signaling and is upregulated in metastatic breast cancer cells but not in normal epithelial mammary cells [67]. Raf kinase inhibitor protein (RKIP) belongs to evolutionarily conserved phosphatidylethanolamine binding protein (PEBP) family and negatively modulates the MAP kinase (MAPK), G protein-coupled receptor kinase-2, and NF-*κ*B signaling cascades [69]. RKIP has been found as a suppressor of PCa metastasis in a mouse model and decreased expression of it is associated with an increased invasive capacity of prostate cancer cells through activation of MEK and ERK [78]. RKIP expression is decreased in PCa, which is associated with increased levels of PSA and PSMA. Missing RKIP expression leads to upregulation of Raf/MEK/ERK and NF-*κ*B (p65/p50) expression, which stimulate PSA and PSMA expression in PCa patients [79, 80]. According to these observations, although restoration of RKIP expression or downstream inhibition of Raf could not affect primary tumor growth, it could inhibit PCa metastasis [81]. In addition, RKIP inhibits invasion, intravasation, and bone metastasis in breast tumor cells through a signaling cascade involving inhibition of MAPK, Myc, and LIN28, causing induction of Let-7 and downregulation of its target genes [82]. BTB-and-CNC homology 1 (BACH1) and high-mobility group AT-hook 2 (HMGA2) expression are inhibited by RKIP signaling pathway via Let-7-dependent mechanism. BACH1 and HMGA2 enhance the development of bone marrow metastatic breast cancer by inducing MMP1, CXCR4, and osteopontin (OPN) gene expression [83]. Let-7 is greatly decreased in breast cancer stem cells [84]. Induction of RKIP expression inhibits the activation of signal transducer and activator of transcription 3 (STAT3), NF-*κ*B pathway, and downstream Yin Yang 1 (YY1) as well as antiapoptotic gene products, causing induction of apoptosis in breast and prostate cancer cells [85–87]. These findings demonstrated that RKIP functions as a suppressor of cancer metastasis, regulates sensitivity to apoptotic stimuli, and can be used as a novel prognostic marker and therapeutic target [86, 88]. Mir-224 expression is significantly upregulated in breast cancer cell lines, which in turn directly inhibits RKIP gene expression [68]. Serum levels of miR-10b are significantly higher in patients with bone metastases relative to patients without bone metastases or the control group [89]. These results can highlight the role of miR-10b as a biomarker for identification of bone metastatic breast cancer or as a marker of prognosis in breast cancer, which requires further studies. An increased serum level of the soluble intracellular adhesion molecule (sICAM1) as well as OCL microRNAs-16 and -378 during OCL differentiation is associated with bone metastasis [71]. MiR-335 is a metastasis suppressor, and SRY-box containing transcription factor SOX4 and Tenascin C (TNC) are among its target genes. The miRNAs -126, -206, and -335 are downregulated in MDA-MB-231 human breast cancer cells [69, 70]. The absence of miR-335 and miR-126 in breast cancer is associated with poor metastasis free survival [69]. Expression study of different miRNAs in various cancers and linking them to any of the factors involved in the development of metastasis can create a new therapeutic target for metastasis.

## 6. New Insight into Metastasis in Breast and Prostate Cancers

Bone metastasis is the most common skeletal complication of malignancies like breast and prostate cancer and is associated with significant morbidity. Recently, a novel molecular mechanism of bone metastasis has been proposed, in which tumor-produced metalloproteinases release EGF to activate the central osteoclastogenic pathway receptor activator of RANKL and promote breast cancer osteolysis [90]. This mechanism includes crucial therapeutic applications that may translate into more effective and site-specific therapies for bone metastases. Metastasis is considered as the ultimate challenge in our efforts to fight against cancer and is the culprit behind most cancer-related deaths. The vast growth in research on metastasis in the past decade has yielded an unprecedented wealth of information on the intrinsic and extrinsic tumor mechanisms determining the metastatic behavior. However, integrating and applying new knowledge-oriented development of metastatic-oriented anticancer drugs are required to thwart the development of metastatic disease at any stage of development [91].

## 7. Discussion 

Metastasis is among the most common causes of death in approximately 90% of patients with solid tumors [92]. “Seed and soil” theory states that the tumor cells (seeds) metastasize to a tissue (soil) in which the conditions for their growth and survival are provided [93]. Bone is among the most common sites of metastasis. Tumor cells, including breast and PCa cells, invade bone through molecular mechanisms and interaction with BM cells. This interaction plays a crucial role in homing of tumor cells to the bone, tumor growth in bone, and increased expression of growth factors required for tumor survival. In this paper, references have been made to this topic. Expression of various factors and markers of metastasis can determine the prognosis of cancer. For example, expression of CD133 mRNA, a marker of bone marrow derived precursor cells, is increased in peripheral blood of patients with metastasis, especially bone metastasis, which seems to be an independent prognostic factor of overall survival [94]. Moreover, the expression of IL-6 in breast cancer is associated with poor prognosis [29]. In addition to prostate and breast cancers, neuroblastoma (NB), which accounts for 10–15% of childhood malignancies, can metastasize to bone and cause osteolytic lesions [95, 96]. NB cells highly express both SDF-1 receptors, that is, CXCR4 and CXCR7. SDF-1/CXCR4 is considered an important signaling pathway for migration and invasion of NB, while SDF-1/CXCR7 is only associated with cell migration [97]. Exposure of NB cells to SDF-1 leads to upregulated expression of integrins like VLA2, VLA3, VLA6, CD56, C-kit, cytokines, and growth factors such as TNF-*α*, vascular-endothelial cell growth factor (VEGF), IL-8, and GM-CSF, which are involved in tumor cell proliferation and survival in BM microenvironment. The majority of NB cells can express the CCR2 chemokine receptor, which reacts with monocyte chemoattractant protein-1 (MCP-1) on OCL and BM stromal and endothelial cells [98]. Galectin-3 binding protein factor is secreted by human NB cells, which stimulates the expression of IL-6 in BM stromal cells during the activation of Erk1/2 pathway. IL-6 also activates OCL [99]. Cytokine-like 1 (CYTL1) positively regulates the proliferation, migration and invasion of NB cells in vitro. There is a direct relationship between CYTL1 and evolution of NB. CYTL1 can be regarded as a factor involved in the growth and metastasis of NB, as a potential therapeutic target, and perhaps as a diagnostic biomarker for NB [100]. Small miRNA molecules play a role in the development of metastasis by targeting important genes involved in different stages of metastasis and can function as antimetastatic or metastamir miRNAs [74].

According to what is described in this review, many factors and signaling pathways are involved in the progression and development of bone metastasis in PCa and breast cancer, understanding the role of which may be useful as new biomarkers for early metastasis detection and eventual improvement of quality of life in patients. For example, ALP and endothelin-1 are two factors increased in osteoblastic metastasis [2, 15] and could be used for metastasis detection. Plasma uPA level is another factor increased in PCa patients [43, 44]; therefore, evaluation of the level of uPA can be a good approach to disease monitoring and prognosis.

Administration of pharmacological inhibitors for these factors and signaling pathways is one of the therapeutic strategies to prevent and/or treat bone metastasis of PCa and breast cancer. This strategy targets the tumor cells as well as the bone microenvironment, so that it can decrease tumor-derived bone lesions. Molecular understanding of metastasis development suggests a protocol in which a combination of target therapy and chemotherapy could delay the onset of bone metastasis, result in disease control, decrease morbidity, and improve survival in patients.

## Figures and Tables

**Table 1:** Tumor cell-derived factors that may affect BM metastasis through interaction with BM microenvironment.

Factors	Function	Expression stimulator	References
(i) Breast cancer cells			
CXCR4	CXCR4 binds to CXCL12 on BM endothelial cell, invades into bone, and causes Akt activation, for which activated Src is required.	ErBb2 signaling increases CXCR4 translation through activation of PI3K/Akt/mTOR pathway.	[11, 21, 22]
VCAM-I	VCAM-1 binds *α*4*β*7 and *α*4*β*1 (VLA-4) integrins on OCL progenitors, causing OCL differentiation and osteoclastogenesis.	VCAM-1 is increased by expressed NF-*κ*B, *α*4, or VCAM-1 blocking antibodies effectively inhibiting bone metastasis.	[23, 24]
CD44	CD44 binding to its receptor (hyaluronan) and its activation lead to IL-8 production in the tumor cell, which stimulates osteolysis.	High levels of CD44 expression on breast cancer cells promote their invasion and adhesion to BM endothelial cells.	[26]
Jagged-1	Jagged-1 by activation Notch signaling stimulates the IL-6 expression in OB; also Jagged-1 expression activates OCL differentiation, and bone resorption occurs.	Jagged-1 expression is again activated by bone-derived TGF-*β* through Smad pathway during osteolytic bone metastasis.	[22, 27–29]
Runx2/CBF*β*	Mediates inhibition of OB differentiation by inducing antagonist of Wnt, sclerostin.	IL-11 and GM-CSF are target genes of Runx2/CBF*β* as OCL activators.	[30]
DKK1	Inhibits OB differentiation, the expression of OPG, and RANKL reduction.	By stimulation of DKK-1 expression in tumor cells, IL-6 inhibits Wnt-mediated osteogenesis, causing an imbalance in bone homeostasis and increased bone degradation.	[17, 31]
CSF-1	The surface form by itself induces the differentiation and survival of OCL, protecting OCL against the inhibitory effect of TGF-*β*.	*⋯*	[32]
PPT-1	It is related to homing, integration, dysfunction in BM microenvironment, and eventual metastasis.	*⋯*	[33]

(ii) Prostate cancer cells			
Endothelin-1	Increases the activity of OB by inhibiting DKK-1 expression by marrow stromal cells; it increases osteoblast expression type 1 collagen.	It is increased in the serum of patients with PCa metastasized to bone.	[15, 34, 35]
CXCR4	Causes tumor cell homing to BM by CXCL12/CXCR4 signaling.	The absence of PTEN and the subsequent activation of PI3K/Akt pathway lead to an increase in CXCR4 expression, regulating the growth and metastasis of bone through CXCL12/CXCR4 pathway.	[20, 36]
Osteonectin	MMP activity, especially MMP2 that is associated with invasion and metastatic potential in cancer cells, is induced by osteonectin.	S-ErbB3 stimulates the bone to secrete osteonectin, which subsequently enhances the invasion of PC-3 PCa cells by interacting with *α*v*β*3 and *α*v*β*5 cell surface receptors.	[37–39]
Shh signaling	PCa cells expressing Shh can directly and specifically induce differentiation in preosteoblasts through a Gli1-dependent mechanism.	Ascorbic acid upregulates paracrine Shh signaling in MC3T3 preosteoblasts. Matrix collagen is formed by OB in presence of AA, potentiating Shh signaling between PCa cells and OBs, inducing OB differentiation.	[29, 40, 41]
TBK1	TBK1 inhibits mTOR signaling pathway, and this inhibition induces dormancy and drug resistance in PCa cells. TBK1 enhances PCa stem-like cells and drug resistance in PCa.	Binding of PCa cell to OB in hematopoietic stem cell niche induces the expression of TBK1.	[42]
u-PA and uPAR	Their expression is associated with aggressive disease phenotype, progression, and metastasis to bone.	Can be used as a factor in prognosis and progression of PCa.	[43, 44]

Abbreviations: CXCR4: C-X-C chemokine receptor 4; CXCL12: C-X-C motif chemokine 12; BM: bone marrow; CXCR4: C-X-C motif receptor type 4; PI3K: phosphoinositide 3-kinase; mTOR: mammalian target of rapamycin; VCAM-1: vascular-endothelial molecule-1; OCL: osteoclast; OB: osteoblast; TGF-*β*: transforming growth factor *β*; Runx2: runt-related transcription factor 2; CBF*β*: core-binding factor subunit beta; GM-CSF: granulocyte macrophage colony stimulating factor; OPG: osteoprotegerin; RANKL: receptor activator of nuclear factor-k*β* ligand; DKK-1: Dickkopf homolog 1; CSF-1: colony stimulating factor-1; PPT-1: preprotachykinin-1; Pca: prostate cancer; PTEN: phosphatase and tensin homolog; MMP: matrix metalloprotease; Shh: Sonic hedgehog; TBK1: TANK binding kinase 1; u-PA: urokinase-type plasminogen activator.

**Table 2:** Role of microRNAs in BM metastasis.

MicroRNAs	Expression	Mechanism of function	Cancer	References
miR-16	Decreased	Inhibits prostate tumor growth through regulation of genes expression such as CDK1 and CDK2	PCa	[61]
miR-141	Increased	Its serum level is increased in patients with bone metastatic PCa and is related to bone metastatic lesion. It has a correlation between ALP levels but not with PSA.	PCa	[62]
miR-143, miR-145	Decreased	Upregulation of them decreases the invasion capacity and EMT. Increased expression inhibits cell viability and colony formation. They suppress tumor sphere formation, expression of CSC markers, and stemness factors such as CD133, CD44, Oct4, C-Myc, and K1f4 in PC-3 cells. HEF1 gene is a target of miR-145.	PCa	[63–65]
miR-203	Decreased	Its reexpression suppresses metastasis and ectopic expression leads to repression of Runx2 and Smad4 expression.	PCa	[66]
miR-218	Increased	Increases the Wnt activity by downregulating its inhibitors SOST, DKK2, and SFRP2 during osteogenesis, which participate in the homing and growth of metastasized cells to the bone. Also it is stimulated in response to Wnt signaling.	BCa	[67]
miR-224	Increased	Inhibits RKIP gene expression.	BCa	[68]
miR-335	Decreased	SOX4 and TNC are among its target genes. Absence of miR-335 and miR-126 in BCa is associated with poor metastasis free survival.	BCa	[69, 70]
miR-16, miR-378	Increased	Serum levels of sICAM1 and OCL microRNAs-16 and -378 which are increased during OCL differentiation, are associated with bone metastasis.	*⋯*	[71]

Abbreviations: CDK: cyclin-dependent kinase; PCa: prostate cancer; BCa: breast cancer; ALP: alkaline phosphatase; PSA: prostate-specific antigen; EMT: epithelial-mesenchymal transition; CSC: cancer stem cell; Oct4: octamer-binding transcription factor 4; HEF1: human enhancer of filamentation 1; Runx2: runt-related transcription factor 2; SOST: sclerostin; DKK2: Dickkopf homolog 1; SFRP2: secreted frizzled related-protein 2; RKIP: Raf kinase inhibitor protein; TNC: Tenascin C; sICAM1: soluble intracellular adhesion molecule; OCL: osteoclast.
